# Analyzing the influence of physical exercise interventions on social skills in children with autism spectrum disorder: insights from meta-analysis

**DOI:** 10.3389/fpsyg.2024.1399902

**Published:** 2024-10-03

**Authors:** Sung Hee Koh

**Affiliations:** Graduate School of Education, Myongji University, Seoul, Republic of Korea

**Keywords:** autism spectrum disorder, ASD, physical exercise intervention, meta-analysis, social skills

## Abstract

**Introduction:**

Children diagnosed with autism spectrum disorder (ASD) commonly encounter difficulties in social interactions and communication, significantly affecting their overall wellbeing. One proposed strategy to address these challenges is through physical exercise interventions. This study aims to conduct a meta-analysis to assess the impact of physical exercise interventions on the social skills of children with ASD.

**Methods:**

To perform this meta-analysis, we followed the Preferred Reporting Items of Systematic Reviews and Meta-Analyses (PRISMA) Statement and the practical guide for transparent reporting of systematic reviews. Eligible studies included randomized controlled trials or quasi-experimental studies investigating the effects of physical exercise interventions on social skills among children with ASD. We used the standardized mean difference (SMD) to measure effect size.

**Results:**

Sixteen studies were included in the meta-analysis. The results indicated a significant improvement in social skills among children with ASD following physical exercise interventions (SMD = −0.54, 95% CI = [−0.63; −0.44]). The moderator analysis underscored the crucial role of age in explaining the intervention outcomes for enhancing social skills, with interventions lasting more than 12 weeks recommended for better social skills improvement.

**Discussion:**

The findings of this meta-analysis provide robust evidence supporting the efficacy of physical exercise interventions in enhancing the social skills of children with ASD. The moderator analysis underscores the importance of considering both the mean age and duration of interventions when implementing such programs. These results underscore the significance of physical exercise as a viable option for improving social skills in this population.

## 1 Introduction

Autism Spectrum Disorder (ASD) is a significant and lifelong neurodevelopmental condition, impacting approximately 27.6 in 1,000 children in the United States (Maenner et al., 2023). It involves noticeable challenges in social skills, coupled with repetitive and restrictive behavior patterns (American Psychiatric Association, 2013). The symptoms associated with ASD can sometimes result in social difficulties, which can make it more challenging for children with ASD (Kunzi, 2015). Given the complex nature of ASD and the lack of universally effective drug treatments, there is a growing need for personalized interventions that cater to the unique needs of individuals within the ASD population. Therefore, this study aims to consolidate the findings of relevant literature on the effectiveness of physical exercise interventions for enhancing social skills in children with ASD.

The Diagnostic and Statistical Manual of Mental Disorders highlights challenges in social skills in children with ASD. ASD can present with variations in social skills, which may include challenges in recognizing and responding to social cues, sustaining conversations, interpreting verbal and nonverbal communication, understanding emotions, and empathizing with others (Watkins et al., 2017). These challenges in adapting to social contexts can result in children with ASD not forming well-established social circles. The social challenge associated with ASD can significantly impact the overall development and wellbeing of affected individuals. Children with ASD may face challenges in establishing emotional connections (Karst and Van Hecke, 2012).

The primary intervention approaches for ASD can be broadly categorized into two groups: drug therapy (Sharma et al., 2018) and behavioral intervention (Haghighi et al., 2023). In treating ASD with medication, psychotropic drugs are commonly used to alleviate symptoms, resulting in a decrease in repetitive behaviors, anxiety, and concentration issues to some extent. However, it is crucial to be aware that these drugs can also come with side effects like drowsiness, dizziness, and loss of appetite. It is important to recognize that the effectiveness of medications can vary from child to child. Behavioral intervention methods encompass structured teaching, Applied Behavior Analysis (Jayousi et al., 2023), Denver Early Intervention Model (Tateno et al., 2021), Sensory Integration Training (Deng et al., 2023), and Developmental, Individual-differences, Relationship-based Floor Time (Rossilli, 2021). However, these methods are typically static and desktop-based, making it challenging to apply them in varied environments.

Physical exercise interventions come with clear benefits. They promote multisensory experiences, facilitate social interaction, contribute to physical fitness and coordination, provide environmental diversity, and support generalization. Moreover, physical exercise interventions are widely recognized for their cost-effectiveness, easy implementation, and minimal adverse reactions, making them a noteworthy option in the quest for effective ASD interventions.

Considering it from a social skills perspective, physical exercise interventions improve executive functions like working memory, inhibition, self-control, and attention, thereby enhancing core symptoms in children with ASD (Howells et al., 2020). However, some studies contradict these findings, suggesting that physical exercise may not have a significant effect on the core symptoms of ASD. For instance, in a randomized controlled trial, Chan et al. (2013) found that physical and mental exercise had no significant effect on the social ability of individuals with ASD.

The inconsistency in these findings may have hindered the decision to use physical exercise as a core symptom intervention for children with ASD. Consequently, a more in-depth analysis is required to specifically and comprehensively assess the impact of physical exercise intervention on the core symptoms of ASD. This will enable the synthesis of existing evidence, leading to more accurate and reliable conclusions.

A thorough and systematic quality assessment of experimental research outcomes through meta-analysis can help address the confusion stemming from inconsistent research results. Some meta-analyses have indicated that physical exercise effectively contributes to the development of operational skills, motor skills, skills-related fitness, social functioning, muscle strength, endurance, social involvement, behavior, communication skills, body awareness, and mental health in individuals with ASD (Healy et al., 2018; Sowa and Meulenbroek, 2012).

While the primary goal is to improve core symptoms, there is a notable gap in research regarding the effectiveness and optimal physical exercise interventions tailored specifically for these core symptoms. This gap encompasses aspects such as intervention duration, type of exercise, characteristics of exercise group, and more. Consequently, this study aims to perform a meta-analysis of existing research evidence on physical exercise interventions targeting the social skills of children with ASD. The objective is to identify the most effective physical exercise intervention strategies and determine areas where emphasis should be placed for maximum benefit. This initiative will serve as the groundwork for designing physical exercise intervention programs aimed at improving the social skills of children with ASD.

## 2 Methodology

To perform this meta-analysis, we followed the Preferred Reporting Items of Systematic Reviews and Meta-Analyses (PRISMA) Statement and the practical guide for transparent reporting of systematic reviews (Page et al., 2021; Sarkis-Onofre et al., 2021).

### 2.1 Study selection and inclusion criteria

In conducting this meta-analysis, a meticulous literature selection process was employed, focusing on research investigating the effectiveness of physical exercise interventions in enhancing social skills among children with ASD. The selection process followed rigorous and systematic procedures, incorporating keyword searches in computerized databases and employing a snowball sampling approach. Comprehensive database searches encompassed platforms such as PubMed, PsycNet, Google Scholar, and Web of Science. To identify pertinent studies, we amalgamated terms in the search string, including “physical exercise,” “physical activity,” “autism spectrum disorder,” “ASD,” “social skills,” and “children.”

### 2.2 Dependent variables

Employing physical exercise intervention among children with ASD can yield diverse benefits, including improvements in social skills. There are a total of 10 sub-factors of social skills as dependent variables used in this study, primarily assessed through the Social Responsiveness Scale (SRS). These sub-factors span dimensions such as social awareness, social cognition, social communication, and social motivation. The 10 sub-factors include autistic mannerisms, social responsiveness, irritability, inappropriate speech, sociability, and social interaction.

### 2.3 Moderating factors

*Gender*: Within the scope of the reviewed studies, there were no studies that exclusively targeted female children. Therefore, in terms of gender, the study was divided into three groups: male-only, mixed, and not specified groups.

*Age of participants*: The mean age of participants was stratified into two distinct groups: those under 7 years old and those aged 7 years old and above.

*Sample size*: The sample size was categorized into three distinct categories: c20 participants, 20–30 participants, and over 30 participants.

*Intervention duration*: The duration of interventions in this study was segmented into two categories: <12 weeks and 12 weeks or more.

### 2.4 Coding methodology

Adhering to established protocols for meta-analytic research, we rigorously crafted our coding procedure to comprehensively capture and quantify essential study attributes and results. Our meticulous coding process systematically extracted 10 pivotal pieces of information from each study. This included details such as authorship, publication year, study setting, design methodology, type of physical exercise employed, intervention duration, participant gender distribution, sample sizes in experimental and control groups, means and standard deviations of intervention effectiveness at pretest and posttest, as well as the geographical location of the study.

### 2.5 Effect size calculations

In this study, we used the standardized mean difference (SMD) as our measure of effect size. We calculated these effect sizes using R-4.3.2 for Windows, which is accessible through r-project.org. This software offers various methods for calculating effect sizes. To understand the significance of these effect sizes, we followed Cohen (1988) guidelines. According to these guidelines, an effect size of 0.80 indicates a large effect, 0.50 suggests a medium effect, and 0.20 implies a small effect.

## 3 Results

### 3.1 Study selection

**Figure 1:**
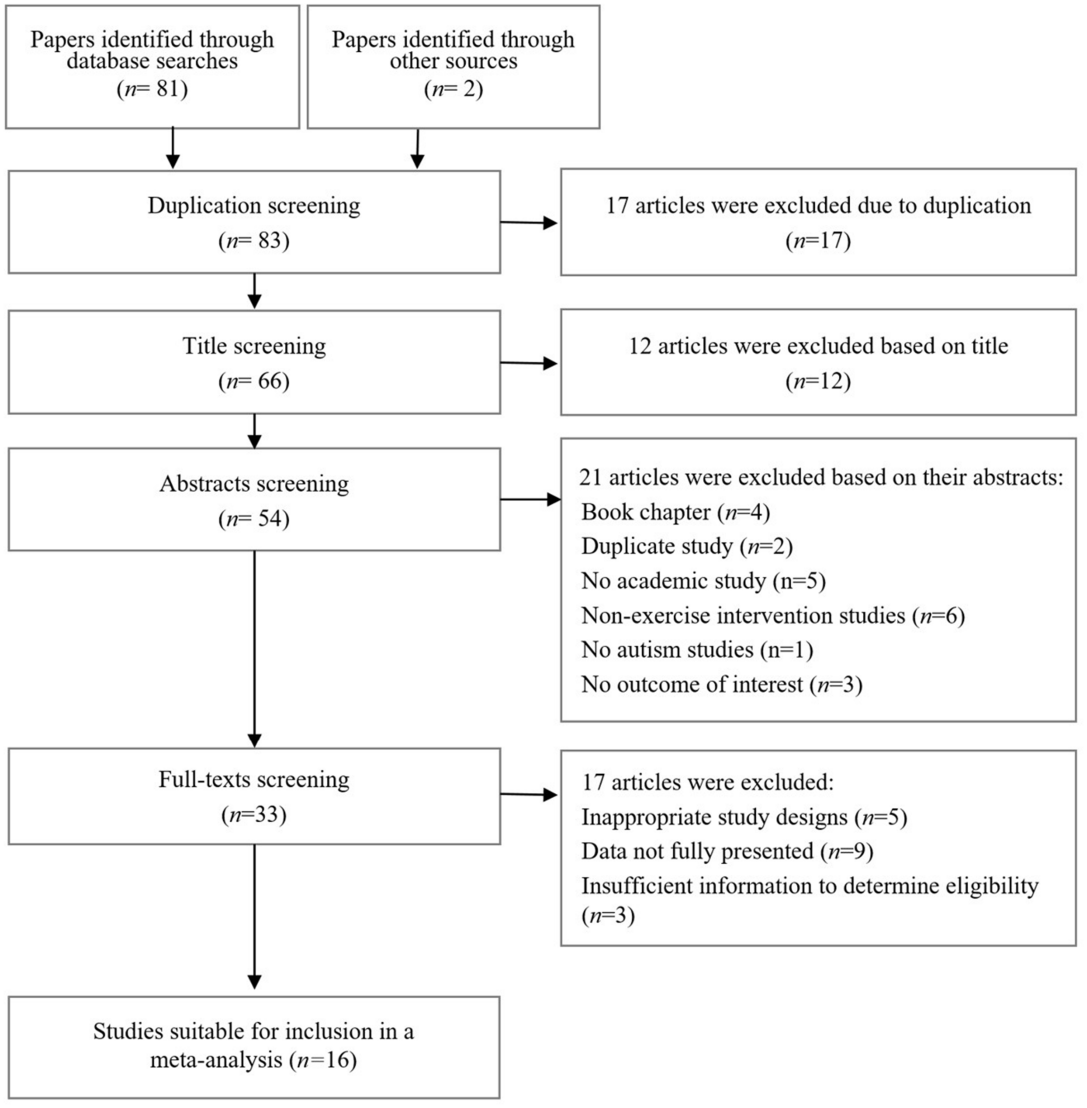
Flowchart of the study selection process according to the PRISMA protocol declarations.

After conducting a database search, a total of 81 documents were initially identified. In addition, two more documents were discovered through snowballing techniques. Upon eliminating duplicates, 66 articles remained. Subsequently, 12 articles were excluded based on title screening. Abstract screening resulted in the exclusion of an additional 21 articles. This left us with 33 articles for full-text screening, with a focus on studies examining the impact of physical exercise interventions on social skills in children with ASD. To ensure methodological rigor, studies lacking the necessary statistical information for calculating effect sizes were excluded from the meta-analysis. Following these criteria, a total of 16 studies, comprising 51 cases, were deemed eligible for inclusion in the meta-analysis (refer to Figure 1 for details).

### 3.2 Assessment of risk of bias

To assess the risk of bias in the included articles, we used the Cochrane Risk of Bias Tool (Higgins and Altman, 2008). This tool assesses each article based on a checklist comprising five items: randomization process, deviation from the intended intervention, missing outcome data, measurement of the outcome, and selection of the reported result. We then categorized each article’s overall bias risk as low risk (indicating low risk across all items), some concerns, and high risk (indicating high risk of bias in at least one domain). Low risk indicates better methodological quality, while high risk suggests a high risk of bias.

**Figure 2:**
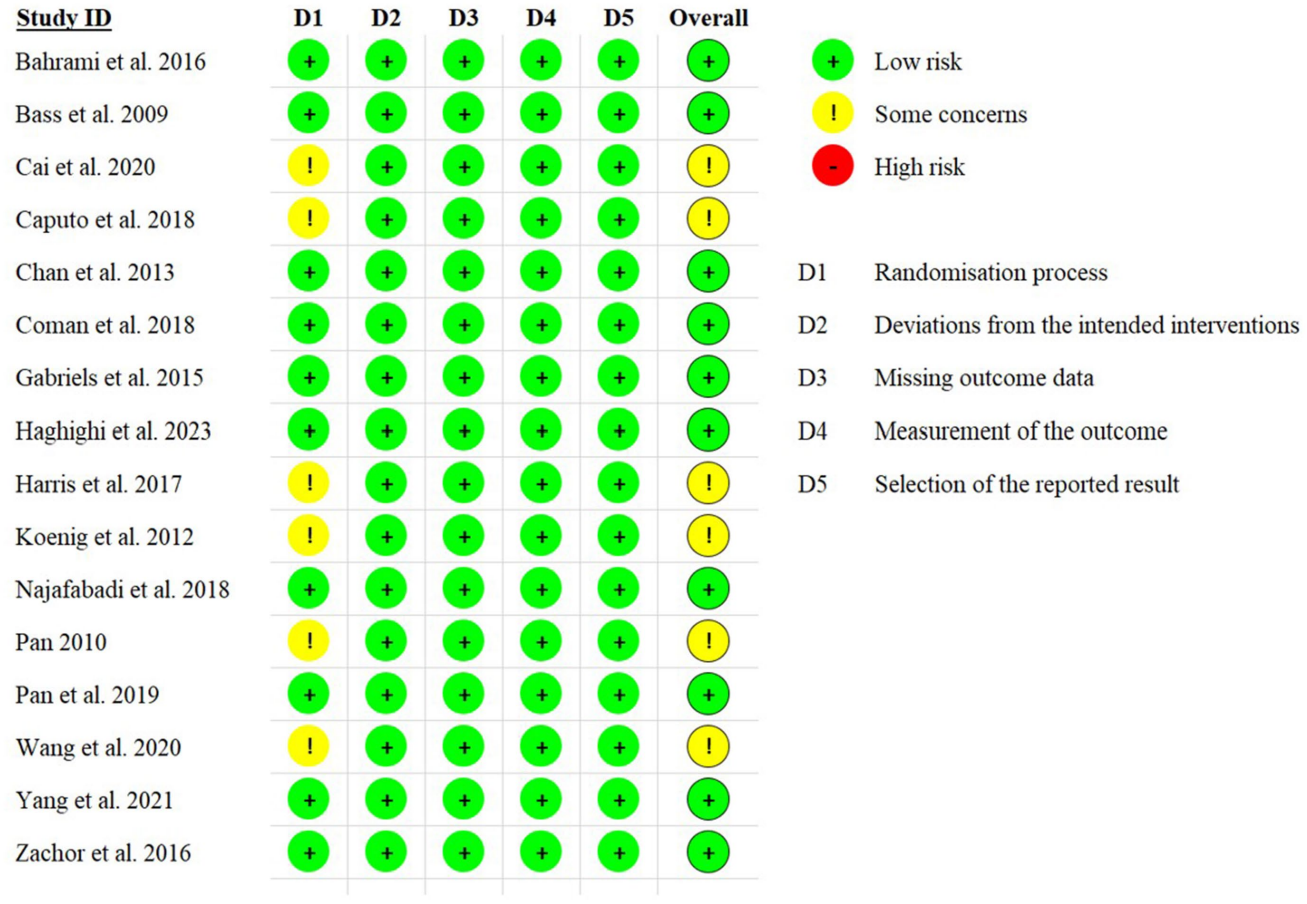
Assessment of risk of bias in the included studies.

Figure 2 presents a visual representation of the risk of bias assessments for each domain of the Cochrane Risk of Bias tool. Among the included articles, 10 (62.5%) were found to have a low overall risk of bias, while 6 (37.5%) showed some concerns regarding the risk of bias. Notably, except for the randomization process domain, the other four checklist items indicated low risk across all 16 articles.

### 3.3 Physical exercise interventions

Among the 16 studies examined, the types of physical exercises reported were diverse. These included horseback riding (*n* = 5), mini-basketball (*n* = 3), swimming (*n* = 2), combined physical training (*n* = 2), karate (*n* = 1), yoga (*n* = 1), mind–body exercise (*n* = 1), and outdoor adventure (*n* = 1).

### 3.4 Social skills

#### 3.4.1 Overall effect size

**Figure 3:**
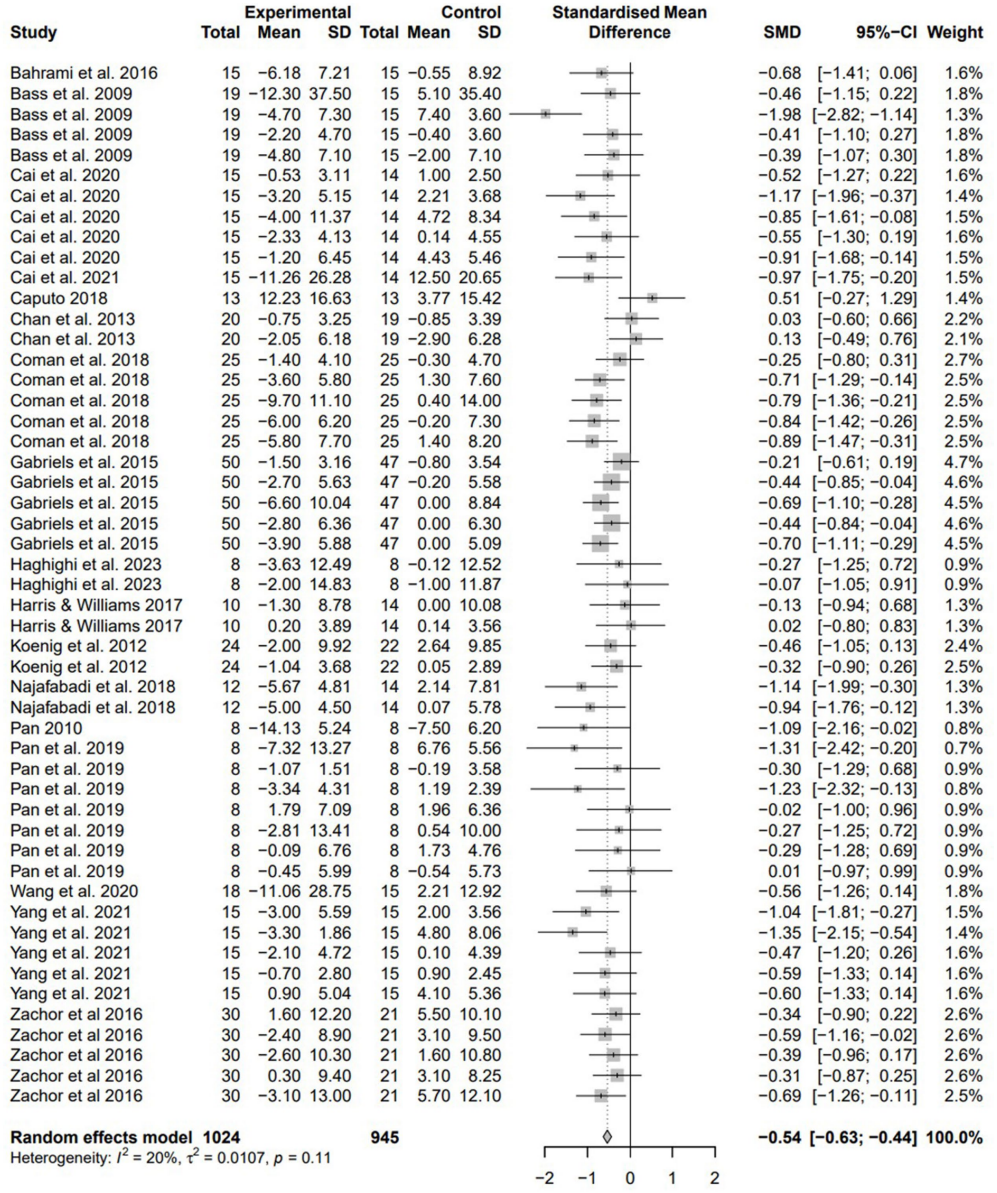
Forest plot of meta-analysis for physical exercise intervention on social skills of children with ASD.

To calculate the mean effect size of physical exercise intervention on the social skills of children with ASD, a total of 51 effect sizes were included, and the results are shown in Figure 3. The heterogeneity of the effect sizes was considered low (I^2^ = 19.8%), so a fixed effect model was used for analysis. The fixed effect model results indicated that the overall mean effect size of physical exercise on social skills was −0.54 (95% CI = [−0.63; −0.44]). Based on Cohen’s (1988) effect size criteria, the effect size of physical exercise on social skills in children with ASD in this study was considered moderate. Furthermore, the 95% confidence interval does not include 0, indicating that the moderate effect measure is statistically significant.

#### 3.4.2 Publication bias

To assess the potential presence of publication bias in our meta-analysis of physical exercise intervention on social skills, we utilized a funnel plot for visual examination, Duval and Tweedie’s trim and fill method, classical fail-safe N, and Egger’s test.

**Figure 4:**
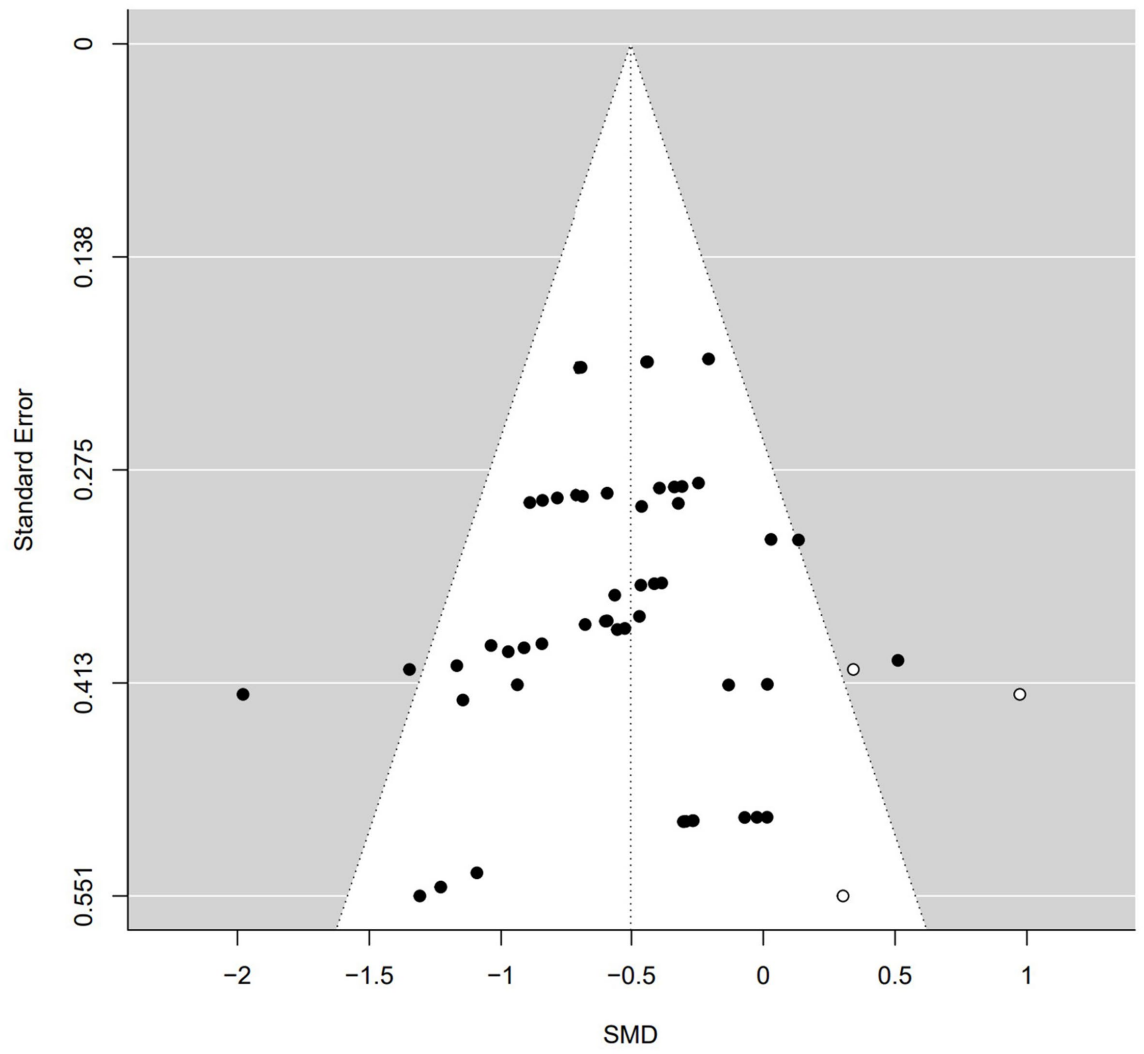
Funnel plot of standard error by SMD.

A funnel plot is illustrated in Figure 4, the horizontal axis is the effect size, and the vertical axis is the standard error. Whether the meta-analysis results have publication bias is determined according to the distribution symmetry of the effect quantity on the Funnel plot test. If the distribution is symmetrical, there is no publication bias; otherwise, there is publication bias (Rothstein et al., 2005). In an ideal scenario without publication bias, data points (depicted as solid circles) from individual case studies would exhibit a symmetrical distribution. Any deviation from this symmetry suggests the potential presence of publication bias. As seen in Figure 4, the distribution of solid circles is slightly left–skewed.

Applying the trim-and-fill method by Duval and Tweedie (2000) reveals that 3 missing studies on the right side are required to achieve symmetry in the funnel plot. The required 3 additional cases are shown on the right as hollow circles in Figure 4.

We also examined publication bias using Rosenthal’s (1979) fail-safe N (Nfs) concept. When Nfs exceeds 5 k + 10, where k represents the number of included case studies, it is unlikely to substantially impact the average effect size. In our study with 51 cases, as long as Nfs is above 265, the meta-analysis results remain stable. Our Nfs value is 2,427, well above the 265 threshold, highlighting the robustness of the meta-analysis. In simpler terms, even if more than 2,427 studies with zero effect size were included, the overall results would stay largely unchanged.

According to the trim-and-fill method by Duval and Tweedie (2000), the absolute value of the adjusted effect size is −0.50 (95% CI = [−0.59; −0.41], *p* = 0.0001), which is smaller than the absolute value of calculated effect size of −0.54 (95% CI = [−0.63; −0.44]).

The results from Egger’s test were not statistically significant (*p* = 0.253), indicating no apparent publication bias. In summary, the moderate contribution of physical exercise to social skills in children with ASD was relatively stable.

### 3.5 Dependent variables

We assessed the impact of physical exercise interventions on the social skills of children with ASD, as measured by the social responsiveness scale (SRS) across dimensions such as social awareness, social cognition, social communication, social motivation, and other dependent variables. The results from a combined meta-analysis of selected studies (see Table 1) reveal a significant moderate effect size (ES = −0.54, 95% CI = [−0.63; −0.44]), indicating a noteworthy improvement in the social skills of children with ASD compared to the control group.

**Table 1:** Effect sizes of dependent variables.

Dependent variable	*n*	Effect size (SMD)	*I*^2^ (%)	95% Confidence interval	
				LL	UL
Overall social skills	51	−0.54	19.8	−0.63	−0.44
Social awareness	7	−0.37	0	−0.60	−0.14
Social cognition	7	−0.82	58	−1.22	−0.42
Social communication	10	−0.52	46	−0.72	−0.31
Social motivation	7	−0.46	0	−0.69	−0.23
Autistic mannerisms	6	−0.72	0	−0.96	−0.47
Social responsiveness	3	−0.64	0	−1.06	−0.23
Irritability	4	−0.58	22	−0.99	−0.18
Inappropriate speech	3	−0.23	0	−0.65	0.20
Sociability	2	−0.37	76	−1.47	0.68
Social interaction	2	−0.64	62	−1.69	0.41

In terms of social skills, social cognition demonstrated a significantly large effect size (ES = −0.82, 95% CI = [−1.22; −0.42]). Meanwhile, social awareness (ES = −0.37, 95% CI = [−0.60; −0.14]), social communication (ES = −0.52, 95% CI = [−0.72; −0.31]), social motivation (ES = −0.46, 95% CI = [−0.69; −0.23]) exhibited a significant moderate effect size. Autistic mannerisms (ES = −0.72, 95% CI = [−0.96; −0.47]), social responsiveness (ES = −0.64, 95% CI = [−1.06; −0.23]), irritability (ES = −0.58, 95% CI = [−0.99; −0.18]) also displayed a significant moderate effect size, while inappropriate speech, sociability, and social interaction did not show significant effect sizes.

### 3.6 Moderator variables

In this section, we examined the impact of physical exercise on the social skills of children with ASD across seven potential moderators. These potential moderators encompassed the impact of physical exercise on social skills concerning study design, gender, mean age of participant group, sample size, length of intervention, type of physical exercise, and study location. Significant differences were found for the mean age of the participant group (*p* = 0.034), while no significant statistical differences were observed in the other moderators: study design (*p* = 0.834), gender (*p* = 0.095), sample size (*p* = 0.798), length of intervention (*p* = 0.070), types of physical exercise (*p* = 0.254), and study location (*p* = 0.827) (refer to Table 2 for details).

**Table 2:** Moderator effects.

Moderators	*F*-test*/ t*-test	*p*	Effect size	*n*	95% CI	
					LL	UL
Total number of studies			−0.54	51	−0.63	−0.44
Study design	*t*(1, 49) = −0.214	0.834				
RCT			−0.53	28	−0.65	−0.41
QE			−0.55	23	−0.69	−0.40
Gender	*F*(2, 48) = 1.686	0.095				
Male			−0.31	3	−0.76	0.42
Mixed			−0.56	46	−0.64	−0.45
Not specified			−1.04	2	−1.63	−0.45
The mean age of the participant group	*t*(1, 49) = −2.176	0.034				
<7 years			−0.65	21	−0.80	−0.50
7 years and greater			−0.46	30	−0.58	−0.35
Sample size	*t*(1, 49) = 0.246	0.798				
<30			−0.54	21	−0.74	−0.35
30 and greater			−0.53	30	−0.64	−0.43
Length of intervention	*t*(1, 49) = 1.812	0.070				
<12 weeks			−0.40	17	−0.55	−0.26
12 weeks and longer			−0.62	34	−0.73	−0.50
Type of physical exercise	*F*(7, 43) = 1.343	0.254				
Horseback riding			−0.54	21	−0.66	−0.41
Mini-basketball			−0.78	12	−1.00	−0.56
Outdoor Adventure			−0.46	5	−0.71	−0.21
Combined exercise			−0.68	4	−1.12	−0.23
Swimming			−0.25	2	−1.82	1.32
Yoga			−0.39	2	−0.80	0.02
Mind–body exercise			0.08	2	−0.36	0.53
Karate			−0.68	1	−1.41	0.06
Location of study	*t*(1, 49) = 0.219	0.827				
United States			−0.55	23	−0.67	−0.42
International			−0.52	28	−0.66	−0.38

#### 3.6.1 Study design

The first question we sought to answer was whether the effectiveness of physical exercise interventions on the social skills of children with ASD was influenced by the study design. Eight studies with 28 cases followed a randomized controlled trial (RCT) design, while nine studies involving 23 cases were designed using quasi-experimental (QE) methods. The results in Table 2 reveal that both RCT and QE studies present a significantly moderate effect size and the difference in the effect sizes between the two categories was not statistically significant [*F*_(1, 48)_ = −0.214, *p* = 0.834].

#### 3.6.2 Gender

As indicated in Table 2, we observed a significant large effect size (ES = −1.04, 95% CI = [−1.63; −0.45]) for the unspecified group. On the other hand, the mixed-gender group, consisting of both male and female children, exhibited a significant moderate effect size (ES = −0.55, 95% CI = [−0.64; −0.45]). However, the effect size (ES = −0.17, 95% CI = [−0.76; 0.42]) was not significant for the male-only group. The differences in the effect sizes among the three gender groups were not statistically significant [*F*_(2, 47)_ = 1.686, *p* = 0.095].

#### 3.6.3 Age

We divided the mean age of the participant group into two categories. The category under 7 years old displayed a significant moderate effect size (ES = −0.65, 95% CI = [−0.8; −0.50]), while the category aged 7 years and older also exhibited a significant moderate effect size (ES = −0.46, 95% CI = [−0.58; −0.35]). A statistically significant difference between the two categories of participants’ age was observed [*F*_(1, 48)_ = −2.176, *p* = 0.034].

#### 3.6.4 Sample size

The sample size was divided into two groups. In the category with over 30 participants, we observed a significant moderate effect size (ES = −0.53, 95% CI = [−0.64; −0.43]). The category with under 30 participants displayed also a significant moderate effect size (ES = −0.54, 95% CI = [−0.74; −0.35]). The difference in the effect sizes between the two groups was not statistically significant [*F*_(1, 48)_ = 0.246, *p* = 0.798].

#### 3.6.5 Length of intervention

We categorized the duration of interventions into two groups. As presented in Table 2, interventions lasting <12 weeks demonstrated a significant moderate effect size (ES = −0.40, 95% CI = [−0.55; −0.26]). Interventions extending for 12 weeks or longer also showed a significant moderate effect size (ES = −0.62, 95% CI = [−0.73; −0.50]). The *t*-test results showed that the difference in the effect sizes between the two categories was not statistically significant [*t*(1, 49) = 1.812, *p* = 0.070]. As a result, this implies that the duration of physical exercise invention does not have an impact on the social skills of children with ASD.

#### 3.6.6 Type of physical exercise

Different types of physical exercises yielded varying results in terms of effect sizes. Specifically, horseback riding (ES = −0.54), mini-basketball (ES = −0.78), outdoor adventure (ES = −0.71), and combined physical training (ES = −0.68) exhibited statistically significant moderate effect sizes. However, swimming, yoga, mind–body exercise, and karate did not show statistical significance. Notably, the differences in the effect sizes among the types of physical exercise were not statistically significant [*F*_(7, 43)_ = 1.343, *p* = 0.254].

### 3.7 Meta-regression analysis

We conducted meta-regression analyses to explore the impact of three moderators (mean age, sample size, and duration in weeks) on social skills. The results indicated that the mean age of participant groups exhibited statistical significance (*p* = 0.016). Consequently, the age of the group moderated the effect of physical exercise on social skills, indicating that younger participants were associated with larger effects (*β* = 0.059, *p* = 0.016), while the other two moderators did not show statistical significance. Thus, the impact of physical exercise on social skills in children with ASD appeared to be influenced by age, while sample size (*p* = 0.809) and intervention duration (*p* = 0.116) did not show significant effects.

## 4 Discussion

This comprehensive meta-analysis encompasses 16 empirical studies involving children diagnosed with ASD between the ages of 3 and 17 years. The primary aim of this meta-analysis was to quantitatively evaluate the effectiveness of physical exercise interventions on social skills in children with ASD. This was achieved by synthesizing various physical exercise intervention programs from the literature within a heterogeneous population. Additionally, the study aimed to explore the influence of several factors, including study design, gender, participant age, sample size, intervention duration, type of exercise, and study location.

The outcomes collectively suggest that physical exercise has a beneficial impact on alleviating social skill challenges in children with ASD. However, it is crucial to acknowledge the considerable variability in outcomes observed across treatments and studies. Notably, physical exercise demonstrated a significant large effect size on social cognition, while social awareness, social communication, and social motivation exhibited a significant moderate effect size.

In our study, we investigated potential sources of variability contributing to significant heterogeneity in the impact of physical exercise on social skills among children with ASD. Utilizing robust variance estimation, we analyzed selected moderator variables. Among children with ASD, the only moderator influencing the impact of physical exercise on social skills was the mean age of the participant group. We divided the participant groups into two categories: those under 7 years old and those over 7 years old. Notably, the effect size was larger in the younger group (mean age < 7 years) compared to the older group (mean age ≥ 7 years). Specifically, physical exercise intervention had a more pronounced effect on preschool children with ASD (ES = −0.65) than on school-age children (ES = −0.46). These findings align with Wang et al. (2023) results, who also observed a stronger effect size in the younger age group. In their study, subgroup analysis based on mean age of the participant group revealed that physical exercise intervention significantly improved ASD core symptoms in children aged 3–6 years (ES = 0.936) compared to those aged 7–12 years (ES = 0.491). The consensus among scholars, including Daniolou et al. (2022) and Yang (2019), supports early behavioral intervention for children with ASD. Notably, research indicates that the brains of preschoolers with ASD exhibit greater plasticity (Calderoni et al., 2016; Dawson and Zanolli, 2003) and malleability (Inguaggiato et al., 2017) during a critical period of character and habit formation. Consequently, the influence of physical exercise intervention is more substantial in preschool children than in school-age children with ASD.

To support the fact that the age of participants acts as a moderator in the relationship between physical exercise and social skills in children with ASD, we conducted a meta-*t* test and a regression analysis. The meta-*t*-test results indicated a statistically significant difference in the impact of physical exercise on social skills between these two categories (t = −2.18, *p* = 0.034). Furthermore, in the regression analysis, where mean age served as the independent variable and the effect size of social skills as the dependent variable, the estimated regression coefficient (*β* = 0.059, *p* = 0.016) was statistically significant. This implies that the mean age of the participant group significantly acted as a moderator, explaining the variance in intervention outcomes related to social skills.

In terms of intervention duration, the study found that social skills improvement was greater for interventions lasting 12 weeks or more (ES = −0.62) compared to those lasting <12 weeks (ES = −0.40). These results align with a study conducted by Wang et al. (2023), which indicated that engaging in physical exercise for more than 12 weeks demonstrated a large effect size (ES = 0.942), whereas participating for <12 weeks resulted in a smaller effect size (ES = 0.195). In summary, a physical exercise duration exceeding 12 weeks had a more substantial impact on improving the social skills of children with ASD compared to a duration of <12 weeks. On the other hand, Tan et al. (2016) reported that the duration of the intervention did not significantly moderate the relationship between exercise and improvement of symptoms. It is crucial to acknowledge that significant outcomes were still achieved in the study with intervention durations of <12 weeks (Gabriel et al., 2015; Pan et al., 2019). However, it remains uncertain whether the effectiveness associated with shorter intervention duration programs persist in the long-term, and what the most effective or ideal intervention duration should be to attain the maximum benefits for social skills in children with ASD. Due to the limited number of studies in the meta-analyses, the examination of intervention duration as a moderator was not completed. When more related studies become sufficiently available, it is necessary to conduct additional analyses on the role of intervention durations as a moderator.

The current investigation also explored how the location of the study might influence the impact of physical exercise on social skills, but found no significant difference between studies conducted in the United States and those conducted internationally. This suggests that the effectiveness of physical exercise interventions may not be influenced by the location of the study. This aligns with the findings of Tarr et al. (2020).

The clinical and research implications of this study are as follows. The findings from this meta-analysis suggest that physical exercise interventions can effectively enhance social skills in children with ASD. These results have significant implications for developing evidence-based interventions for this population. Furthermore, the study indicates that the participant group’s mean age significantly influences the impact of physical exercise on social skills. Specifically, physical exercise interventions are more effective in improving social skills among preschool children than school-age children with ASD. These findings suggest that interventions may need to be tailored to the child’s age to maximize their effectiveness.

## 5 Conclusion

This study aimed to validate the effectiveness of physical exercise interventions in improving social skills in children with ASD. The findings suggest several key points. Firstly, physical exercise had a significant positive impact on the social skills of children with ASD, indicating improvement following the interventions. Moreover, the examination of potential moderators influencing these outcomes revealed that the mean age of the participant group played a crucial role in explaining the variation in intervention outcomes. Interestingly, potential moderators such as study design, gender, sample size, type of physical exercise, and study location did not seem to significantly influence the effectiveness of the interventions on social skills.

In summary, physical exercise emerges as an effective strategy for enhancing social skills, especially beneficial for younger children under the age of 7, and interventions lasting more than 12 weeks are recommended for optimal results. These findings underscore the role of physical exercise as an evidence-based intervention option.

Nevertheless, it is important to acknowledge the limitations of this meta-analysis. Firstly, many studies reviewed did not specify whether participants were simultaneously using medication or undergoing other forms of intervention alongside exercise regimens during the study period, potentially introducing confounding variables. Secondly, the inclusion of studies covering individuals across a wide spectrum of ASD severity levels suggests that the findings might not be universally applicable to all subpopulations. Therefore, caution is warranted when generalizing these results. Thirdly, it is important to acknowledge that the absence of including children’s IQ as a moderating factor in this study represents a clear limitation. For future research, it is essential to consider a broader range of moderating factors, including IQ. This will enable a more comprehensive analysis and contribute to the existing body of knowledge on the topic. Lastly, the review focused solely on immediate post-intervention effects, highlighting the necessity for longitudinal studies to explore the long-term benefits of physical exercise interventions for children with ASD comprehensively.

## Data Availability

The original contributions presented in the study are included in the article/supplementary material, further inquiries can be directed to the corresponding author.
